# Three Level Thoracolumbar Spondylectomy for Recurrent Giant Cell Tumour of the Spine: A Case Report

**DOI:** 10.5704/MOJ.1811.013

**Published:** 2018-11

**Authors:** NA Faruk, MZ Mohd-Amin, DN Awang-Ojep, YY Teo, CC Wong

**Affiliations:** Department of Orthopaedics, Sarawak General Hospital, Kuching, Malaysia; *Department of Orthopaedics, Universiti Malaysia Sarawak, Kota Samarahan, Malaysia; **Department of Pathology, Universiti Malaysia Sarawak, Kota Samarahan, Malaysia

**Keywords:** spinal giant cell tumour, en bloc spondylectomy, vertebrectomy, dural tear, massive blood loss

## Abstract

Giant cell tumour (GCT) is a benign tumour but can be locally aggressive and with the potential to metastasise especially to the lungs. Successful treatments have been reported for long bone lesions; however, optimal surgical and medical treatment for spinal and sacral lesions are not well established. In treating spinal GCTs, the aim is to achieve complete tumour excision, restore spinal stability and decompress the neural tissues. The ideal surgical procedure is an en bloc spondylectomy or vertebrectomy, where all tumour cells are removed as recurrence is closely related to the extent of initial surgical excision. However, such a surgery has a high complication rate, such as dura tear and massive blood loss. We report a patient with a missed pathological fracture of T12 treated initially with a posterior subtraction osteotomy, who had recurrence three years after the index surgery and subsequently underwent a three level vertebrectomy and posterior spinal fusion.

## Introduction

Giant cell tumour (GCT) of the bone is rare in the vertebrae above the sacrum. The prevalence ranges from 1.4 to 9.4% of total bone GCT’s^1^. Management of these tumours is challenging as there is a high recurrence rate especially when the tumour is not removed in entirety. However, recent literature suggests that a complete removal should be performed for GCT’s of the spine and radiotherapy reserved for inoperable tumours or those where complete tumour resection was not possible^2^.

## Case Report

We present a case of a 25 year-old man who complained of worsening back pain and left lower limb weakness and radiculopathy for two weeks. He was unable to walk due to the pain and weakness. There was no bowel or bladder incontinence but he had loss of appetite and significant weight loss.

He had history of fall and sustained a stable burst fracture of T12. He was treated with an extension body cast at that time as there was no suspicious lesion on the radiographs. During follow-up, he developed a kyphotic deformity of which we performed pedicle subtraction osteotomy of T12 a year after the primary injury. He defaulted the follow-up after surgery.

**Fig. 1:**
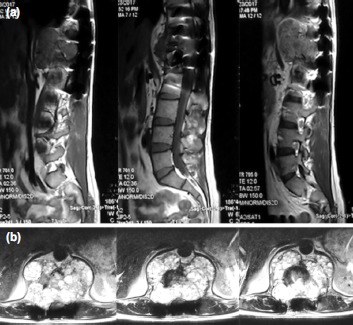
(a) Pre-operative MRI sagittal view (T1 Weighted - from left to right) showing the mass extending anteriorly involving T11,T12 and L1. (b) Pre-operative MRI axial view (T2 Weighted) at the level of T12 showing an expansile mass surrounding the T12 vertebrae.

Examination revealed a posterior midline surgical scar measuring 12cm. There was a mild kyphotic deformity. His hip and knee flexion were weak with a medical research council (MRC) muscle power grading of 3. The ankle and toes had MRC muscle power grading of zero. Magnetic resonance imaging (MRI) was suggestive of an aggressive spinal tumour over T12 with extension to T11 and L1 (Fig. 1). Computed tomography of the lungs revealed no lung metastasis.

**Fig. 2:**
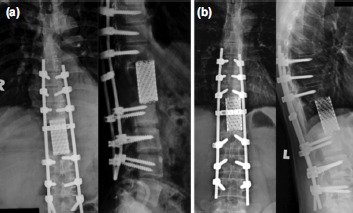
(a) Immediate post-operative radiograph. (b) One year post-operative radiograph showing no loosening of implants.

He underwent posterior extension of fusion from T8-L4 with total vertebrectomy of T11, T12 and L1. Excision of the posterior elements of T11 and L1 then removal of the pedicles of T11 and L1 was done. *En bloc* tumour removal was attempted but scarring and adhesions to the diaphragm prevented an *en bloc* removal, so piecemeal vertebrectomy of T11, T12 and L1 and excision of tumour was performed. Three segmental arteries were ligated on the right side to facilitate cage insertion and the bone gap reconstructed with a titanium mesh cage filled with bone cement (Fig. 2). The reconstructed mesh was shorter than the total height of the removed vertebral bodies, as we shortened the spinal column, but not exceeding one vertebral body and two discs height to prevent cord buckling. Intra-operatively there was adhesions due to the previous scarring causing dura and diaphragm tear during excision of the tumour, which we could not repair. A dura sealing agent was used to seal the tears. He lost 7.7 litres of blood during the surgery requiring massive blood transfusion.

**Fig. 3:**
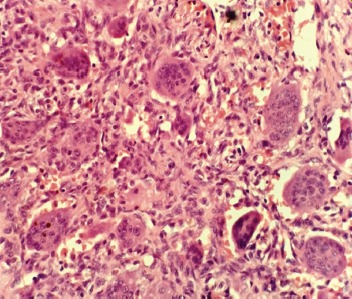
Photo-micrograph of the tumour showing monotonous spindle cells amidst many osteoclastic giant cells (hematoxyline and eosin stain, x40).

Post-operatively, his neurological deficit improved to MRC muscle grade 5 and he was able to walk with a single crutch with minimal back pain. The wound healed well. At the last follow-up at one and a half years post-operatively, there was complete neurological recovery with good functional outcome. Histopathological examination confirmed the diagnosis of GCT of the spine (Fig. 3).

## Discussion

Surgical treatment of choice for GCT is wide excision or *en bloc* spondylectomy to achieve good oncological control to prevent local recurrence. However, because of the proximity of vital structures such as the spinal cord, dural sac, nerve roots and large blood vessels, intralesional or marginal resection may be a safer option because of the risk of fatality or morbidity as a result of the aggressive surgical technique.

Dura tear is a known surgical complication in complex spinal surgery. Luzzati reported a series of 13 out of 38 cases operated for spondylectomy which had intra-operative dura tear with three requiring revision surgery for the leak. He had 14 major complications and 22 minor complication in his series^3^. Post-operative complications following dura tear include postural headache, nausea, vomiting, pain or tightness in the neck or back, dizziness, diplopia due to VI cranial nerve paresis and tinnitus. Potential serious complications are CSF fistula formation, pseudomeningocele, meningitis, arachnoiditis and epidural abscess^3^.

In our case, the dura tear was because of adhesion and scarring from the previous surgery. Post-operatively the patient had severe headache that required complete rest in bed for two weeks. Patients who had durotomy intra-operatively and repaired primarily have no difference in clinical outcome^4^. Primary ‘watertight’ closure was the best treatment. Additional fibrin glue, augmentation by muscle, fat or graft can be an option when the closure is suboptimal^4^. These may be sutured directly to the defect or applied in an “on-lay” fashion. Insertion of a drainage tube was not associated with higher rates of revision surgery. However, suction drainage was found to be a significant risk factor for revision surgery.

The patient also had poor oxygenation post-operatively requiring low dose supplemental oxygen for one week post-operatively. However, he responded well to physiotherapy and supportive oxygen. Resection of the vertebral body creates a defect on the medial part of the diaphragm thus compromising lung function postoperatively. He did not develop any haemothorax or pneumothorax post-operatively.

The patient had massive blood loss requiring blood transfusion. Several interventions had been used in an attempt to reduce blood loss during surgery for spinal tumours. Pre-operative embolisation of spinal metastasis to reduce intra-operative blood has been recommended. Hypotensive anaesthesia is commonly implemented to reduce blood loss but post-operative visual loss has been reported^5^. Bednar *et al* reported their experience using tranexamic acid to minimise operative blood loss but the finding was not statistically significant^5^. In this case, intra-operatively we used adrenaline-soaked gauze and thrombin-soaked gel foam to control local bleeding.

Multiple level spondylectomy is a challenging and complex surgery with possibility of multiple complications intra-operatively. Haemostatic agents such as thrombin should be made available in anticipation of bleeding in cases of tumour excision. Dura sealing agents should also be available as revision surgeries have higher incidence of iatrogenic dura tear. Suspicious bony lesions even with prior history of trauma should be biopsied to avoid missing a pathological fracture and delayed treatment.

In our patient, despite intraoperative problems, the surgery was rewarding and the patient had excellent neurological recovery, good functional outcome and is currently disease-free at one year follow-up.
